# Plasma extracellular vesicle proteins are associated with stress-induced myocardial ischemia in women presenting with chest pain

**DOI:** 10.1038/s41598-020-69297-0

**Published:** 2020-07-23

**Authors:** Mirthe Dekker, Farahnaz Waissi, Joelle van Bennekom, Max J. M. Silvis, Nathalie Timmerman, Ingrid E. M. Bank, Joan E. Walter, Christian Mueller, A. H. Schoneveld, Raymond M. Schiffelers, Gerard Pasterkamp, Diederick E. Grobbee, Robbert J. de Winter, A. Mosterd, Dominique P. V. de Kleijn, Leo Timmers

**Affiliations:** 1 Department of Vascular Surgery, University Medical Centre, Utrecht, the Netherlands; 2 Department of Cardiology, Academic Medical Centre, Amsterdam, the Netherlands; 3 Department of Cardiology, University Medical Centre, Utrecht, the Netherlands; 4 Department of Cardiology, St. Antonius Hospital, Nieuwegein, the Netherlands; 5 Department of Cardiology and Cardiovascular Research Institute Basel (CRIB), University Hospital Basel, University of Basel, Basel, Switzerland; 6 Department of Clinical Chemistry and Haematology, University Medical Centre, Utrecht, the Netherlands; 7 Julius Center for Health Sciences and Primary Care, University Medical Centre, Utrecht, the Netherlands; 8 Department of Cardiology, Meander Medical Centre, Amersfoort, the Netherlands; 9 Netherlands Heart Institute, Utrecht, the Netherlands; 10 Department of Cardiology, UMC Utrecht, Heidelberglaan 100, 3508GA Utrecht, the Netherlands

**Keywords:** Biochemistry, Cell biology, Biomarkers, Cardiology, Risk factors

## Abstract

Diagnosing stable ischemic heart disease (IHD) is challenging, especially in females. Currently, no blood test is available. Plasma extracellular vesicles (EV) are emerging as potential biomarker source. We therefore aimed to identify stress induced ischemia due to stable IHD with plasma extracellular vesicle protein levels in chest pain patients. We analyzed 450 patients suspected for stable IHD who were referred for ^82^Rb PET/CT in the outpatient clinic. Blood samples were collected before PET/CT and plasma EVs were isolated in 3 plasma subfractions named: TEX, HDL, LDL. In total 6 proteins were quantified in each of these subfractions using immuno-bead assays. CD14 and CystatinC protein levels were independent significant predictors of stress-induced ischemia in the LDL and the HDL subfraction and SerpinC1 and SerpinG1 protein levels in the HDL fraction. Subgroup-analysis on sex revealed that these associations were completely attributed to the associations in women. None of the significant EV proteins remained significant in men. Plasma EV proteins levels are associated with the presence of stable IHD in females presenting with chest pain. This finding, if confirmed in larger cohort studies could be a crucial step in improving diagnostic assessment of women with suspected IHD.

## Introduction

Ischemic heart disease (IHD) remains one of the most common causes of death worldwide^1^. IHD comprises two most prevalent clinical syndromes: acute coronary syndrome (ACS) and stable angina/stable IHD. ACS can be quickly diagnosed in most cases by either ST elevation on ECG or elevated troponin levels. Diagnosing stable IHD is more complex with a wide variety of non-invasive tests^1,2^. Although imaging modalities provide reasonable sensitivity and specificity (80–90%) they become more and more subject of debate because of high costs, radiation exposure and increasing use in inappropriate low-risk patients^3,4^. A recently performed cost-effectiveness analysis of non-invasive imaging showed a prevalence of obstructive coronary artery disease (CAD) in only 25% of suspected stable IHD patients^5^. Another concern that merit consideration in IHD is the evolving knowledge regarding sex differences in pathophysiology, symptoms, diagnostic test performance, and prognosis. Women less frequently have obstructive CAD, yet higher mortality rates compared to men within equal age range^6–8^. Since only 25% of patients with suspected IHD appear to have IHD, the need for a novel blood based biomarker to detect stable IHD is evident.

Plasma extracellular vesicles (EVs) are relatively unexplored as biomarker source. EVs have a bilipid membrane layer. Vesicles are ~ 50–1000 nm in size and include exosomes, micro vesicles and micro particles^9^. EVs can be produced by any cell type and consist of proteins, mRNA, miRNA and lipid particles derived from the cell of origin. EVs contain bioactive content that may influence (patho)physiological processes^10,11^. Previous studies showed associations between EV protein levels and future cardiovascular risk^12,13^. It is, however, unknown whether specific EV proteins could be used as biomarker to diagnose stable IHD. We therefore investigated if plasma EV protein levels in 3 subfractions are associated with stress-induced ischemia, in patients presenting with chest pain in the outpatient clinic.

## Materials and methods

### Study population

**Figure 1:**
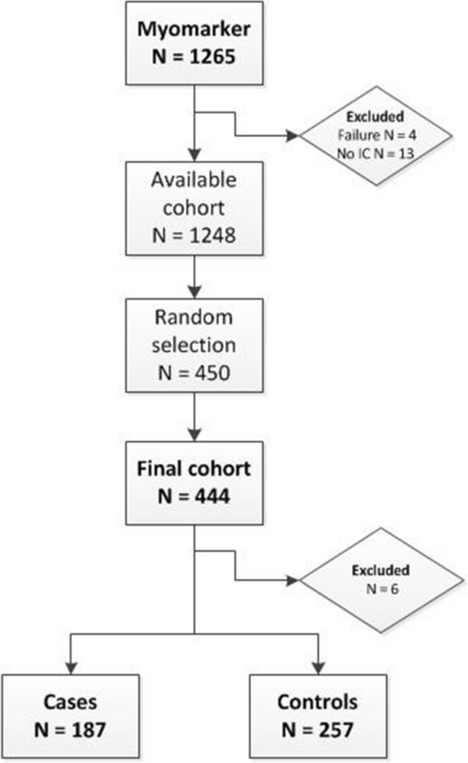
Flowchart patient selection. Original data from myomarker cohort. *IC* informed consent.

The MYOMARKER (MYOcardial ischaemia detection by circulating bioMARKERS) study is a prospective single-centre observational cohort study of consecutively enrolled patients (> 18 years) with suspected CAD who presented at the outpatient clinic of the Meander Medical Centre (Amersfoort, the Netherlands) between August 2014 and September 2016. All patients underwent a Rubidium-82 PET/CT. The complete cohort consists of 1,265 patients. For the purpose of this study a random sample of 450 patients was selected for the analysis (Fig. 1). The study (NL5078) was approved by the Medical Ethics Committee-United (MEC_U) and performed in accordance with the Declaration of Helsinki. Written informed consent was obtained from all participants.

### Primary outcome

Primary outcome of the study was stress-induced ischemia. The adjudication of the presence of the primary outcome was based on the results of both myocardial perfusion imaging (MPI), and coronary angiography (CAG) data if available. Rubidium-82 PET/CT MPI results were assessed according to the 17-segment model of the American Heart Association^14^. All scans were evaluated by 2 experienced observers. In short, the summed difference score (SDS) was the total difference between the stress and rest score for each of the 17 segments. Cases (patients with stable IHD) were defined as patients with SDS score ≥ 2 and visual agreement by both observers. Patients were considered as control if their SDS score was < 2. Based on a previously performed, comparable study, we decided to add available CAG data to the MPI results to improve the diagnostic accuracy of MPI^15^. CAG images were interpreted with quantitative coronary angiography (QCA) by 2 experienced clinicians using Cardiovascular Angiography Analysis System software (CAAS 7.3, Pie Medical Imaging, Maastricht, The Netherlands). CAG data was available in 146 patients. In total 27 (6%) patients were reclassified from stress-induced ischemia to no ischemia and 11 (2%) patients were reclassified from no ischemia into having stress-induced ischemia.

### Identification of proteins

Previously performed proteomics analyses was used to select the proteins^12,16^. Selected proteins were Serpin C1 (SC1), CD14, Serpin G1 (SG1), Cystatin C (CC), Plasminogen (PLG) and Serpin F2 (F2). Protein levels were determined in in blood plasma and in all three EV subfractions.

### Isolation of extracellular subfractions

Venous blood was collected in EDTA tubes directly before MPI from the peripheral intravenous cannula. Blood tubes were centrifuged 10 min at 1850×*g* at room temperature (RT) within 30 min after collection. Plasma was aliquoted and directly stored at − 80 °C. Plasma extracellular vesicle subfractions were isolated using a modified protocol based on the publication of Burstein et al.^17^. Detailed description of the isolation protocol used can be found in the supplemental materials. In short, a subset of EVs co-precipitated with Low-Density Lipid particles (LDL) while others co-precipitate with High-Density Lipid particles (HDL), which allows separation. In addition, one subfraction is analysed without the LDL and HDL subfractionation and therefore referred to as TEX subfraction. For the sequential isolation of the subfractions Dextran Sulphate (DS) (MP Biomedicals), Manganese (II) Chloride (MnCl2) (Sigma-Aldrich) solutions and Xtractt buffer (1:4) (Cavadis BV) were used (Supplemental Fig. 1).

### Characterization of extracellular vesicles

Both the modified protocol which was used as well as extracellular vesicle characterization are described in detail in two previously published paper (especially in the supplemental materials of Zhang et al.)^16,18^. In short, we used density gradient centrifugation of the 3 plasma subfractions, all density fractions were characterized by CD9 western blot analysis as EV specific antibody. Lipid particles were identified with ApoB in all density gradient fractions. The presence of EVs was confirmed also visually with electron microscopy (EM) showing the typical bilayer EVs separated from lipid particles. The proteins studied in this manuscript (SC1, CD14, SG1, PLG, CC and SF2) were shown in the density gradient fractions that were shown with CD9 western blotting and EM, and, absent in the density gradient fractions with lipid particles. To get easy access to these data an EV-track ID was created: EV200044, in which the data is structured in a uniform way as suggested by Sluijter et al.^19^.

Additional to the previous performed experiments to characterize EVs in all three subfractions, we performed a size characterization analysis using Nanoparticle Tracking Analyzer (NTA) (supplemental materials and supplemental Fig. 2). This showed relatively small EVs in the TEX fraction (mean 84 nm), slightly larger EVs in the LDL fraction (mean 101 nm), and the largest particles in the HDL fraction (mean 120 nm). All three fraction show much larger particles then LDL (22–29 nm) or HDL (7–12 nm) particles^20^.

### Quantification of EV protein levels

EV concentrations of selected proteins were measured using the Bio-plex 200 systems (Bio-Rad). Briefly, magnetic Magplex-C Microspheres (Luminex) were conjugated with antibodies specific for the proteins of interest. 50 μl of the protein lysates from the EV subfractions were added to the bead-antibody complexes and were incubated for 45 min. Subsequently, biotin-labelled antibodies were added and again incubated for 45 min. Dilution of the biotinylated antibodies differed for each of the six proteins, respectively; SG1 1:200, SF2 1:400, SC1 1:100, CC 1:200, PLG 1:400 and for CD14 1:400. Bio-Plex 200 system was used for the sample analysis and calibrated each day before use, in accordance to the specifics of the manufacturer. Streptavidin–phycoerythrin (1:500) (Moss) was used to quantify the concentration. Calibration lines were created for each of the six proteins using recombinant proteins (supplemental Fig. 3). Washing steps were executed with a Hydrospeed plate washer (Tecan). SC1 and CD14 biotinylated antibodies were diluted in assay buffer 1x (Thermo Fischer Scientific) with human anti mouse antibody (HAMA) blocking reagent (1:90) (Fitzgerald). All other dilutions were prepared in assay buffer 1x. Data analysis was performed using Bio-Plex Manager Software version 6.1.1 (Bio-Rad). Antibodies and recombinant proteins are listed in supplementary Table 1. Protein concentration were measured in pg/mL. Non-specific binding of the detection antibodies to the bead was verified as follows: capture antibodies of the six proteins were conjugated to six different beads (CD14 + Bead 77; SC1 + Bead 57; PLG + Bead 67; SF2 + Bead68; CC + Bead 27 and SG1 + Bead 55) and mixed. This beadmix was incubated with one of the recombinant protein (the amount was depended on the protein: CD14 and SF2 = 2222 pg/ml; or SC1, PLG and SG1 = 8333 pg/ml; or CC = 5555 pg/ml) in six wells for each recombinant protein (total 36wells). In each of the six wells with the same recombinant protein one biotinylated detection antibody of the six proteins was added. No non-specific binding of the detection antibodies was found. A signal was only seen in the containing the correct combination of recombinant protein with the detection antibody for that specific protein, while other wells had a value comparable to the blanc (supplemental Table 2).

### Statistical analysis

Continuous data are expressed as mean ± standard deviation or median ± interquartile range, categorical data as frequencies with corresponding percentages. Differences in continuous variables were compared by independent t-test or Mann–Whitney were appropriate. Dichotomous variables were compared by Chi-square or Fisher’s exact test were appropriate. All EV proteins were standardized with the use of synthetic liposomes (SVs) to serve as internal control (detailed description can be found in the supplemental materials). To provide insight in raw data we provided baseline levels of EV proteins as measured with Bio-Plex.

Distribution of all EV proteins were visually inspected with boxplots and histograms. Because of the skewed distribution of all proteins, we logarithmically transformed them to achieve normal distributions. After transformation all distributions were visually inspected again. The SV standardized and logarithmically transformed variables were used for the logistic regression analyses. All EV proteins were tested in a univariable logistic regression model as well as in a multivariable model adjusted for known cardiovascular risk factors (sex, age, smoking, hypertension, hypercholesterolemia, diabetes mellitus and coronary artery disease). Additional adjustment was performed for cardiovascular medication separately.

We performed exploratory subgroup analyses based on sex and history of coronary revascularization, either percutaneously or with coronary bypass surgery. All subgroup analyses were adjusted for known cardiovascular risk factor (were possible). All hypotheses tests were two-sided with a critical significance level of < 0.05. We did not correct for multiplicity in our study because of the clear exploratory nature of this study, as suggested by Rothman^21^. Statistical analysis was performed with R software (R software, version 3.5.1).

## Results

In total 444 out of the total 450 patients were analysed. Baseline characteristics are summarized in Table 1. Sex and generally accepted risk factors were equally distributed between cases and controls. Patients with stable IHD were slightly older compared to patients without stable IHD (67.65 vs. 69.47, p value 0.043). History of CAD and previous complaints of angina were more common among cases (respectively 69.5% vs 37.0%, p value < 0.001 and 58.8% vs. 35.4%, p value < 0.001).

**Table 1:** Baseline characteristics.

	**Control**	**Case**	**P value**
n	257	187	
**Demographics**			
Age	67.65 (9.10)	69.47 (9.63)	0.043
%Women	65 (25.3)	46 (24.6)	0.956
BMI	27.47 (4.42)	27.18 (4.49)	0.508
**Previous history**			
Cardiovascular disease	237 (92.2)	176 (94.1)	0.557
Coronary artery disease	95 (37.0)	130 (69.5)	< 0.001
Acute myocardial infarction	54 (21.0)	87 (46.5)	< 0.001
Coronary revascularization	93 (36.2)	112 (59.9)	< 0.001
Angina pectoris	91 (35.4)	110 (58.8)	< 0.001
Kidney disease	4 (3.9)	6 (8.1)	0.393
**Risk factor**			
Smoking	37 (14.5)	38 (20.4)	0.127
Diabetes Mellitus	55 (21.4)	47 (25.1)	0.419
Hypertension	171 (66.5)	120 (64.2)	0.677
Hypercholesterolemia	151 (59.0)	113 (60.4)	0.835
Family history CAD	75 (29.5)	54 (29.3)	1.000
**Medication**			
Platelet inhibitors	149 (58.0)	136 (72.7)	0.002
Oral anticoagulants	50 (19.5)	42 (22.5)	0.514
Blood pressure lowering agents	209 (84.6)	170 (92.9)	0.013
Lipid-lowering agents	166 (67.2)	139 (76.0)	0.062

Values are displayed as mean ± SD or frequency (%), Case = patient with a SDS score ≥ 2 on myocardial perfusion imaging (MPI), and/or functionally relevant coronary artery disease on coronary angiogram. *CAD* Coronary artery disease. *CVD* history of CAD or peripheral vascular disease or history of ischemia CVA, Kidney disease = eGFR < 30.

Raw EV-biomarker levels are shown for cases and controls (supplemental table 3). Univariable analysis showed significant differences between cases and controls for SC1 HDL (odds ratio (OR) 1.31, 95% CI: 1.04–1.66), CD14 HDL (OR 1.46, 95 CI: 1.02–2.11), CD14 LDL (OR 1.54, 95% CI: 1.09–2.16), SG1 HDL (OR 1.37, 95% CI: 1.04–1.80), CC HDL (OR 1.35, 95% CI: 1.04–1.76), CC LDL (OR 1.70, 95% CI: 1.17–2.46). Adjusted for both cardiovascular risk factors and additional for cardiovascular medication, all biomarkers with statistically significant impact in the unadjusted analysis remained significant. Results can be found next to the univariable analysis in Table 2. As can be seen in supplemental Fig. 4, none of the selected proteins measured in whole plasma would have the ability to distinguish between cases and controls.

**Table 2:** Logistic regression analysis for stress-induced myocardial ischemia.

Biomarker	Unadjusted		RF adjusted^a^		RF + Med Adjusted^b^	
	OR (95% CI)	P value	OR (95% CI)	P value	OR (95% CI)	P value
Serpin C1 HDL	1.31 (1.04–1.66)	0.024	1.38 (1.05–1.80)	0.017	1.39 (1.06–1.82)	0.017
Serpin C1 LDL	1.35 (0.91–2.01)	0.135	1.33 (0.86–2.05)	0.204	1.29 (0.83–2.01)	0.252
Serpin C1 TEX	0.87 (0.52–1.44)	0.581	0.79 (0.46–1.37)	0.400	0.80 (0.46–1.39)	0.428
CD14 HDL	1.46 (1.01–2.11)	0.042	1.56 (1.04–2.35)	0.032	1.62 (1.07–2.45)	0.023
CD14 LDL	1.54 (1.09–2.16)	0.014	1.62 (1.11–2.35)	0.013	1.64 (1.12–2.41)	0.011
CD14 TEX	1.52 (0.83–2.78)	0.172	1.43 (0.74–2.75)	0.288	1.50 (0.77–2.92)	0.230
Serpin G1 HDL	1.37 (1.04–1.80)	0.027	1.42 (1.05–1.92)	0.024	1.41 (1.04–1.92)	0.028
Serpin G1 LDL	1.04 (0.76–1.43)	0.810	1.24 (0.87–1.75)	0.230	1.27 (0.90–1.81)	0.179
Serpin G1 TEX	1.08 (0.68–1.73)	0.737	1.27 (0.74–2.17)	0.393	1.25 (0.72–2.19)	0.432
Cystatin C HDL	1.35 (1.04–1.76)	0.026	1.38 (1.03–1.85)	0.034	1.41 (1.04–1.91)	0.025
Cystatin C LDL	1.70 (1.17–2.46)	0.006	1.69 (1.11–2.55)	0.014	1.70 (1.12–2.60)	0.014
Cystatin C TEX	1.45 (0.81–2.58)	0.210	1.14 (0.59–2.21)	0.694	1.13 (0.58–2.20)	0.726
Serpin F2 HDL	1.00 (0.82–1.22)	0.967	1.07 (0.87–1.32)	0.519	1.07 (0.87–1.32)	0.521
Serpin F2 LDL	0.99 (0.82–1.21)	0.945	1.03 (0.84–1.27)	0.772	1.02 (0.83–1.26)	0.825
Serpin F2 TEX	0.89 (0.63–1.24)	0.480	0.92 (0.63–1.30)	0.582	0.94 (0.65–1.36)	0.738
Plasminogen LDL	1.16 (0.78–1.71)	0.468	1.28 (0.83–1.96)	0.262	1.32 (0.85–2.04)	0.212
Plasminogen TEX	1.02 (0.63–1.64)	0.950	0.92 (0.55–1.54)	0.755	0.94 (0.56–1.57)	0.804

Biomarker levels are log-transformed and standardized per synthetic vesicle and shown as mean ± SD. Original assay units are pg/ml.

*RF* Risk factor, *Med* medication.

^a^RF adjusted; age, sex, hypertension, hypercholesterolemia, smoking, diabetes mellitus and coronary artery disease.

^b^RF + Med adjusted: platelet inhibitors and blood pressure lowering agents.

**Figure 2:**
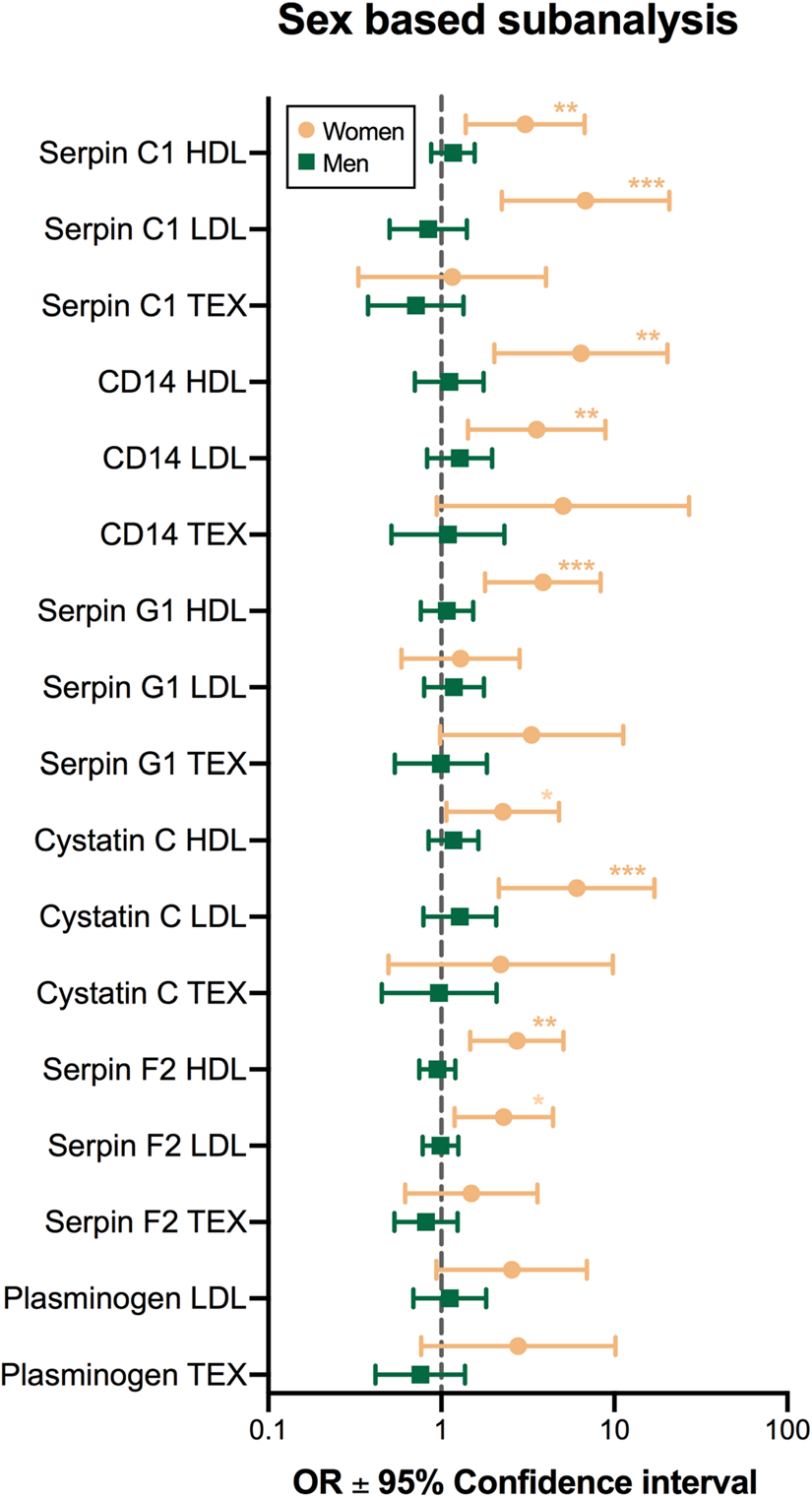
Forestplot of multivariable logistic regression subanalysis stratified on sex. Horizontal bars indicate adjusted* odds ratios and corresponding 95% CI for ischemia. Biomarker levels are log-transformed and standardized per synthetic vesicle. Original assay units are pg/ml. *Adjusted for: age, hypertension, smoking, hypercholesterolemia, diabetes mellitus and coronary artery disease.

We performed an exploratory adjusted subgroup analysis based on sex. Raw baseline EV biomarker levels were stratified on sex are provided in the supplemental data (supplemental table 4). Figure 2 shows the OR with corresponding confidence intervals for all EV biomarker levels stratified on sex. In addition to the proteins with significant impact for the complete study population three additional proteins reach significance among women. All proteins became insignificant among men. Supplemental Fig. 5 shows an additional exploratory subgroup analysis on previous coronary revascularization. Most important differences were found between patients who underwent a coronary artery bypass graft (CABG). Consistent with the results from the complete study cohort SC1 HDL, CD14 LDL and CC LDL remained significant adjusted predictors of stress-induced ischemia.

## Discussion

We showed that EV protein levels in plasma subfractions differ between patients with and without stress-induced ischemia. Exploratory subgroup analysis revealed that the differences in biomarker levels were only seen in women. This finding emphasizes the difference between men and women and the need for sex-specific diagnostic strategies. The role of previous revascularization also needs consideration. Subgroup analysis on CABG imply that EV proteins might have a limited role in CABG patients. This could be the result of the complicated interpretation of MPI results in CABG patients, however, it is an important finding which should be considered for future studies.

Biomarkers to detect stress-induced ischemia are studied extensively. However, only few biomarkers have shown some evidence as potential biomarker. Best known are; high-sensitive cardiac troponin (hs-cTn) and BNP^22–24^. Both have shown significantly higher blood levels in patients with myocardial ischemia compared to those without. Nevertheless, their diagnostic performance remains limited. Differences in EV protein levels depict differences upon cell-level. EV content might change already in a very early stage of the disease, whereas hs-cTn and BNP are end-products of cell damage. This difference might be the explanation for the great potential of plasma EV proteins as diagnostic biomarker. Larger studies are needed to build a reliable plasma EV-based biomarker model to test whether EV proteins are able to improve clinical decision making. However, the differences of protein levels in women observed in this cohort are remarkable and may lead directly to improved patient care for women.

### EV proteins levels and ischemic heart disease

Serpin C1, known as anti-thrombin is an anticoagulant protein. Its main function is inhibition of thrombus formation^25^. In our study plasma EV SC1 in the HDL subfraction was associated with stress-induced ischemia. Only one, however not comparable, previously performed study was done on plasma EV SC1, this study showed no effect of statin therapy on plasma EV protein levels of SC1^26^. CD14 is a membrane anchored protein known from its function in the innate immune system as TLR4 co-receptor^27^. Both the HDL and LDL fractionated plasma EV CD14 protein was associated with stress-induced ischemia. This is in line with previous studies on plasma EVs where CD14′s role in thrombotic and inflammatory processes during CVD was shown^12,18,28^.

Serpin G1, also known as C1-inhibitor, is an acute phase protein which regulates the complement activation^29,30^. Its main function is the inhibition of coagulation and atherosclerotic plaque formation^31^. In our study population plasma EV Serpin G1 in the HDL subfraction was associated with stress-induced ischemia. Previous research on plasma EV content in cardiovascular disease showed a strong correlation between Serpin G1 and low-grade inflammation, which is known as keystone in atherosclerotic disease^28,32^.

Cystatin C is an inhibitor of proteases that play a key role in inflammation. It is produced and secreted by cardiomyocytes and its synthesis is elevated when the myocardium experiences ischemia^33^. Plasma Cystatin C is known as an important marker for renal dysfunction and also for its close relationship with CAD^34–36^. De Hoog et al. showed that plasma EV-Cystatin C was associated with an acute coronary syndrome in the TEX subfraction in male patients^37^. Interestingly we also found an association of EV CC but only in the HDL and LDL subfractions. ACS significantly differs pathophysiologically from stable IHD which might explain this difference. It might also be that plasma EV protein concentrations differ between EV subfractions depending on the atherosclerotic burden and plaque stability. Future studies should provide more insight in this.

Serpin F2, known as alpha-2-antiplasmin, is a protease inhibitor and best known from its function in inhibition of plasmin, which has an important role in fibrinolysis ^38,39^. The sex based subgroup analysis revealed F2 in both LDL and HDL subfraction as adjusted significant predictor for stress-induced ischemia. Plasminogen is known as precursor of plasmin, which plays a role in fibrinolysis. No association of PLG with stress-induced ischemia was found. To our knowledge only one study has been performed on EV-plasminogen, which looked into the effect of statin use on plasma EV-plasminogen levels. They found an strong association with EV-plasminogen levels after statin treatment^26^.

### Sex differences

Our exploratory subgroup analysis on sex showed the differences in biomarker levels between cases and controls were only seen in women. Sex differences in CVD risk are well known, but not well understood^6^. It has been proposed that inflammation, metabolic syndrome and adiposity contribute more significantly to the pathophysiology of CVD in females compared with males^40^. Our results contribute to this statement since plasma EV-Cystatin C, CD14 and Serpin G1 are known to be important proteins within the inflammatory cascade. This was also found in a previous study on EVs which showed their relation with obesity and metabolic complications^28^. The same was seen in 2 studies about the relation between plasma inflammatory markers and the development of CVD in women^41,42^. Recently, E. Lau et al. published an article on sex difference in biomarkers within the CVD field^43^. They performed a large study on > 7,000 patients (54% female) and examined in total 71 proteins, of which 61 differed between men and women. The sex differences observed in our study are in line with these results. The associations found in women could be a crucial step in improving the diagnostic assessment of ischemia in this subgroup. However, these findings should be interpreted with caution and seen as hypothesis generating since this is based on a subgroup analysis with low numbers. Future studies with large numbers are needed to test this hypothesis and explore the role of an EV based model improves clinical care.

### Strengths and limitations

This study was a retrospective single centre analysis with myocardial perfusion analysis as reference standard for stable IHD. Since patients were included in the study after referral for perfusion imaging, indicating relative high suspicion, there will be referral and selection bias. This study has a relatively small sample size and results from subgroup analysis could only be interpreted as hypothesis generating. There are several strengths of this study. The cohort consists of real-world data in a centre with high number of outpatient clinic visits and myocardial perfusion imaging.

## Conclusion

We showed associations between EV protein levels and stress-induced ischemia. Subgroup analysis on sex showed that all significant associations were completely attributed to women and none of them remained significant in men. Larger studies are needed to confirm our findings, but EV proteins should be considered as promising future tool to improve the diagnostic process for women with suspected stable IHD.

## Supplementary information

Supplementary file1

## Data Availability

The datasets generated during and/or analysed during the current study are available from the corresponding author on reasonable request.

## Abbreviations

IHD: Ischemic heart disease
EV: Extracellular vesicle
ACS: Acute coronary syndrome
CAD: Coronary artery disease
MPI: Myocardial perfusion imaging
CAG: Coronary angiography
SDS: Summed Difference Score
CABG: Coronary artery bypass graft
PCI: Percutaneous coronary intervention
