# Supplementary material for: Plasma extracellular vesicle proteins are associated with stress-induced myocardial ischemia in women presenting with chest pain
Source: Sci Rep. 2020 Jul 23;10:12257. doi: 10.1038/s41598-020-69297-0 (PMC7378184; doi:10.1038/s41598-020-69297-0)
Supplement: Supplementary file 1 — Supplementary file1 [file 41598_2020_69297_MOESM1_ESM.docx]

Plasma extracellular vesicle proteins are associated with stress-induced myocardial ischemia in women presenting with chest pain.
Mirthe Dekker^a,b*ᶧ^; Farahnaz Waissi^a,bᶧ^; Joelle van Bennekom^a^; Max J.M. Silvis^c^; Nathalie Timmerman^a^*;* Ingrid E.M. Bank^d^; Joan E.Walter^e^, Christian Mueller^e^, A.H. Schoneveld^f^; Raymond M. Schiffelers^f^, Gerard Pasterkamp^f^*;* Diederick E. Grobbee^g^; Robbert J. de Winter^b^; A. Mosterd^h^; Dominique P.V. de Kleijn^a,i^; Leo Timmers^d^

^a^Department of Vascular Surgery, University Medical Centre Utrecht, the Netherlands

^b^Department of Cardiology, Academic Medical Centre, Amsterdam, the Netherlands

^c^Department of Cardiology, University Medical Centre Utrecht, the Netherlands

^d^Department of Cardiology, St. Antonius hospital Nieuwegein, the Netherlands

^e^Department of Cardiology and Cardiovascular Research Institute Basel (CRIB), University Hospital Basel, University of Basel

^f^Department of Clinical Chemistry and Haematology, University Medical Centre Utrecht, the Netherlands

^g^Julius Center for Health Sciences and Primary Care, University Medical Centre Utrecht, the Netherlands

^h^Department of Cardiology, Meander Medical Centre Amersfoort, the Netherlands

^i^Netherlands Heart Institute, Utrecht, the Netherlands

**Address for correspondence:**
*Drs. Mirthe Dekker
Address: Department of Cardiology UMC Utrecht Heidelberglaan 100 3508GA Utrecht

Phone: 088-7550458 fax: -

Email: [m.dekker-17@umcutrecht.nl](mailto:m.dekker-17@umcutrecht.nl)

Word count: 5427 (including references), 4182 (excluding references)

**Supplemental materials**

Isolation of extracellular vesicle plasma subfractions

LDL and HDL subfractions can be obtained from plasma by a DS and MnCl2 solution of DS: 0.05%, MnCl2: 0.05M and DS: 0.65%, MnCl2: 0.2M, respectively. For LDL subfraction isolation, 25uL plasma was diluted in 80uL phosphate buffered saline (PBS) (Gibco), followed by addition of 5µL magnetic beads (Nanomag®-D plain, 130mm (1:25) (Micromod)). 15µL of a standard amount of synthetic liposomes, coated with DSG-PEG2000 (Nanocs) and fluorescently labeled with 18:1 liss rhod pe (Merck), was added to each plasma sample to be able to correct for loss of the pellet during isolation. DS and MnCl2 were added into the total volume of 125 μL and were mixed. The mixture was incubated 5 min at room temperature (RT). Subsequently, the samples were placed on a bio-plex handheld magnet (Bio-Rad) and incubated 15 min at RT. The pellets were lysed with 125µL Roche complete lysis-M with protease inhibitors (Roche). To remove magnetic beads and other debris, samples were centrifugated at 3200xg, 10 min. Fluorescence of the synthetic liposomes were measured with SpectraMax® Multi-Mode Microplate reader (Molecular Devices) directly after completion of the isolation protocol. For HDL isolation, the protocol is repeated when using 115µL supernatant above the LDL pellet. For the TEX subfraction, 25µL plasma was diluted in 80µL PBS, 5µL Nano-mag®-D PEG-OH (1:25) (Micromod) and 15µL of the synthetic fluorescent labelled liposomes. Xtractt buffer was added and the samples were mixed. The protocol is repeated following LDL isolation procedure. The pellet is used as TEX subfraction.

Nanoparticle tracking analyzer

Microparticles were characterized with the Nanoparticle Tracking Analyzer (NTA) N200 (Malvern Panalytical). Directly after EV isolation, samples were resuspended in PBS since a dilution factor was required to be measurable on the NTA (10^7-9^ particles/mL). Data about particle size distribution and concentration were collected for 3 times 30s at room temperature by the NTA Software. Results are shown in supplemental figure 2.

**Supplemental tables**

| **Supplemental table 1. Antibodies and recombinant proteins to detect selected proteins** | |
| --- | --- |
| **Detected protein** | **Recombinant/antibody used** |
| CD14 | - Recombinant human CD14 protein (R&D systems, #383-cd, Minneapolis, MN, USA) - Anti-human CD14 (R&D systems, #MAB3822) - Biotin labelled anti human CD14 (R&D systems, #BAF383) |
| Serpin C1 | - Recombinant human Serpin C1 (R&D systems, #1267-PI) - Anti-human Serpin C1 (Novus Biologicals, #NBP1-05149, Centennial, CO, USA) - Biotin labelled anti-human Serpin C1 (R&D systems, #BAF1267) |
| Serpin G1 | - Recombinant human Serpin G1 (R&D systems, #2488-pi) - Anti-human Serpin G1 (R&D, #MAB2488) - Biotin labelled anti-human Serpin G1 (R&D systems, #BAF2488) |
| Serpin F2 | - Recombinant human Serpin F2 (R&D systems, #1470-pi) - Anti-human Serpin F2 (R&D systems, #MAB1470) - Biotin labelled anti-human Serpin F2 (R&D systems, #BAF1470) |
| Plasminogen | - Recombinant human Plasminogen (Sunny Lab, #P20401, Maryland, MD, USA ) - Anti-human Plasminogen (Hytest, #8F11, Turku, Finland) - Biotin labelled anti-human plasminogen (Novus Biologicals, #NB120-10174B); |
| Cystatin C | - Recombinant human Cystatin C (R&D systems, #1196-PI-010) - Anti-human Cystatin C (R&D systems, #MAB11962) - Biotin labelled anti-human Cystatin C (R&D systems, #BAM11961) |

| **Supplemental table 2. Non-specific binding of detection antibodies to beads** | | | | | | |
| --- | --- | --- | --- | --- | --- | --- |
|  | **Protein** |  |  |  |  |  |
| **Bead+recombinant** | Serpin G1 | Serpin F2 | CD14 | Serpin C1 | Cystatin C | Plasminogen |
| BM+rCD14 | 252 | 162 | 15221 | 347 | 114 | 235 |
| BM+rSerpin C1 | 248 | 174 | 407 | 2175 | 124 | 239 |
| BM+rSerpin G1 | 1435 | 170 | 431 | 364 | 127 | 246 |
| BM+rSerpin F2 | 236 | 8598 | 422 | 347 | 125 | 247 |
| BM+rPlasminogen | 242 | 346 | 459 | 341 | 135 | 7341 |
| BM+rCystatin C | 239 | 171 | 415 | 347 | 12115 | 244 |
| BM+Blanc | 236 | 178 | 418 | 352 | 138 | 246 |
| BM = Bead Mix; r=Recombinant; Data are given in fluorescence determined in Bioplex 200 | | | | | | |

| **Supplemental Table 3. Baseline table biomarker levels** | | |
| --- | --- | --- |
|  | **Controls** | **Cases** |
| n | 257 | 187 |
| **Biomarker** |  |  |
| Serpin C1 HDL | 1599700 [874794-2436300] | 1615100 [1026650-2880050] |
| Serpin C1 LDL | 7286400 [6227300-8461500] | 7031600 [6271400-8438850] |
| Serpin C1 TEX | 63651 [51552-79938] | 61126 [51632-80071] |
| CD14 HDL | 6394 [5257-8023] | 6515 [5116-8221] |
| CD14 LDL | 21558 [17208-27288] | 22888 [18223-29183] |
| CD14 TEX | 24205 [20172-28387] | 25530 [21525-31592] |
| Serpin G1 HDL | 1698300 [1250400-2506500] | 1837500 [1363750-2454850] |
| Serpin G1 LDL | 2217900 [1296400-4020300] | 1986700 [1316450-3629000] |
| Serpin G1 TEX | 155625 [123842-194833] | 159038 [127929-203080] |
| Cystatin C HDL | 1653 [1199-2699] | 1802 [1312-2779] |
| Cystatin C LDL | 13909 [11638-16432] | 15178 [12498-18131] |
| Cystatin C TEX | 44703 [37928-52203] | 45561 [38521-57165] |
| Serpin F2 HDL | 171786 [113306-218560] | 158221 [107755-209066] |
| Serpin F2 LDL | 16518 [11137-24230] | 16694 [11567-23348] |
| Serpin F2 TEX | 182840 [120703-257491] | 180735 [126682-263633] |
| Plasminogen LDL | 460528 [367072-570831] | 453136 [353731-565912] |
| Plasminogen TEX | 638970 [543764-792345] | 652140 [553731-792734] |
| Raw biomarkers levels in pg/ml. Values are shown as median ± IQR. Case = patient with a SDS score ≥ 2 on myocardial perfusion imaging (MPI), and/or functionally relevant coronary artery disease on coronary angiogram | | |

| **Supplemental Table 4. Sex specific baseline biomarker levels** | | | | | | |
| --- | --- | --- | --- | --- | --- | --- |
|  | **Men control** | **Men case** |  | **Women control** | **Women case** |  |
| n | 192 | 141 |  | 65 | 46 |  |
| **Biomarker** |  |  |  |  |  |  |
| Serpin C1 HDL | 1470150 [758809-2297725] | 1510200 [916266-2413800] |  | 1942700 [1446500-2688500] | 2186800 [1254875-3232275] |  |
| Serpin C1 LDL | 7470250 [6318025-8646225] | 7020600 [6149700-8423600] |  | 6831100 [6003300-8029000] | 7208350 [6442550-8574875] |  |
| Serpin C1 TEX | 60637 [50790-78036] | 59831 [50950-75966] |  | 67434 [52571-83018] | 66142 [54088-85369] |  |
| CD14 HDL | 6151 [5107-7741] | 6234 [4969-7832] |  | 7015 [5775-8817] | 7362 [6240-9402] |  |
| CD14 LDL | 20754 [17155-25077] | 22098 [17825-27389] |  | 24311[19240-30468] | 25415 [20721-30893] |  |
| CD14 TEX | 23762 [19820-27651] | 24787 [20778-31180] |  | 26239 [21017-29567] | 27032 [23258-32670] |  |
| Serpin G1 HDL | 1677650 [1205000-2558575] | 1802500 [1310400-2302300] |  | 1710900 [1335000-2352500] | 2184700 [1630000-3157600] |  |
| Serpin G1 LDL | 2022150 [1184150-3553525] | 1986700 [1372100-3540100] |  | 2571300 [1715600-4828600] | 2012050 [1121575-3831525] |  |
| Serpin G1 TEX | 153714 [123521-192380] | 154287 [121473-204311] |  | 167343 [127631-204509] | 175613 [137774-201934] |  |
| Cystatin C HDL | 1587 [1173-2637] | 1773 [1243-2707] |  | 1940 [1287-2897] | 1914 [1485-3140] |  |
| Cystatin C LDL | 14018 [11868-16293] | 15203 [12471-18253] |  | 13562 [11437-16653] | 15101 [12605-17992] |  |
| Cystatin C TEX | 44195 [37549-52912] | 45444 [37887-57252] |  | 45088 [38791-51440] | 46801 [41711-56144] |  |
| Serpin F2 HDL | 176112 [113692-221701] | 158576 [105260-197965] |  | 164757 [113306-206687] | 158205 [124351-228865] |  |
| Serpin F2 LDL | 16827 [11188-25148] | 16766 [11180-22879] |  | 15649 [11079-22302] | 16392 [12473-26074] |  |
| Serpin F2 TEX | 182596 [119966-257389] | 173786 [123599-257779] |  | 201913 [133497-257491] | 197794 [144200-275583] |  |
| Plasminogen LDL | 466211 [368607-571308] | 461961 [366585-591729] |  | 449127 [362876-560086] | 414538 [296258-482069] |  |
| Plasminogen TEX | 632888 [535633-778020] | 642386 [539671-781082] |  | 671643 [569467-822916] | 688261 [594694-841418] |  |
| Raw biomarkers levels in pg/ml stratified on sex. Values are shown as median ± IQR. Case = patient with a SDS score ≥ 2 on myocardial perfusion imaging (MPI), and/or functionally relevant coronary artery disease on coronary angiogram | | | | | |  |

**Supplemental figures**


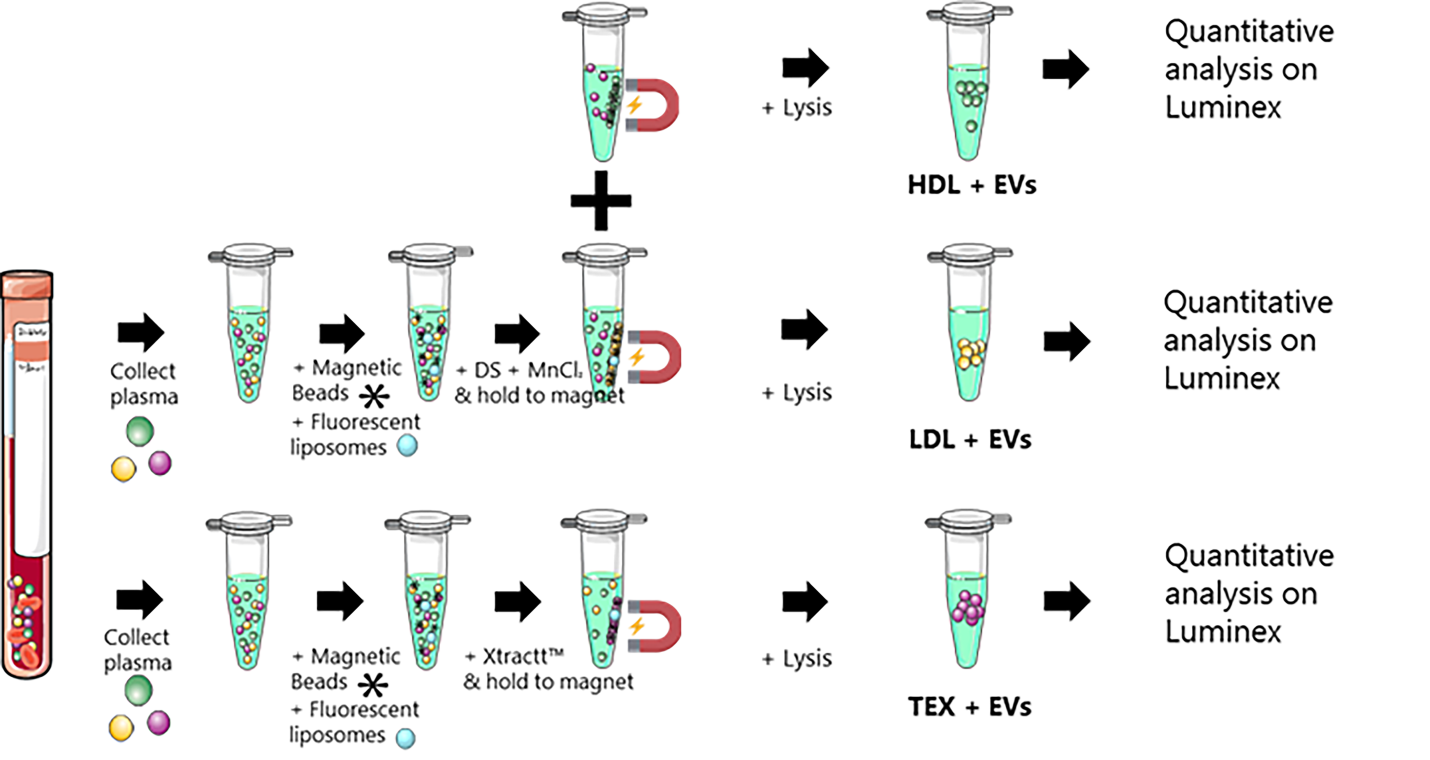


**Supplemental Figure 1**
Sequential isolation of plasma subfractions and sequential lysis and analysis of extracellular vesicles.

**
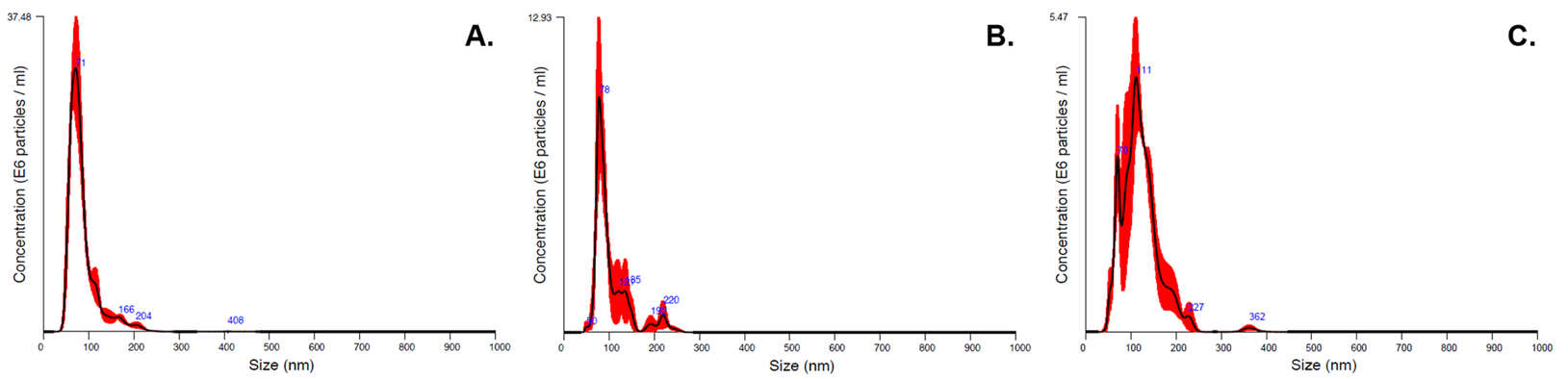
**

**Supplemental figure 2. Nanoparticle tracker analyzer.** Size distribution from NTA measurements in
A. TEX subfraction, B. LDL subfraction and C. HDL subfraction. Distributiondata are summarized from three measurements.

**
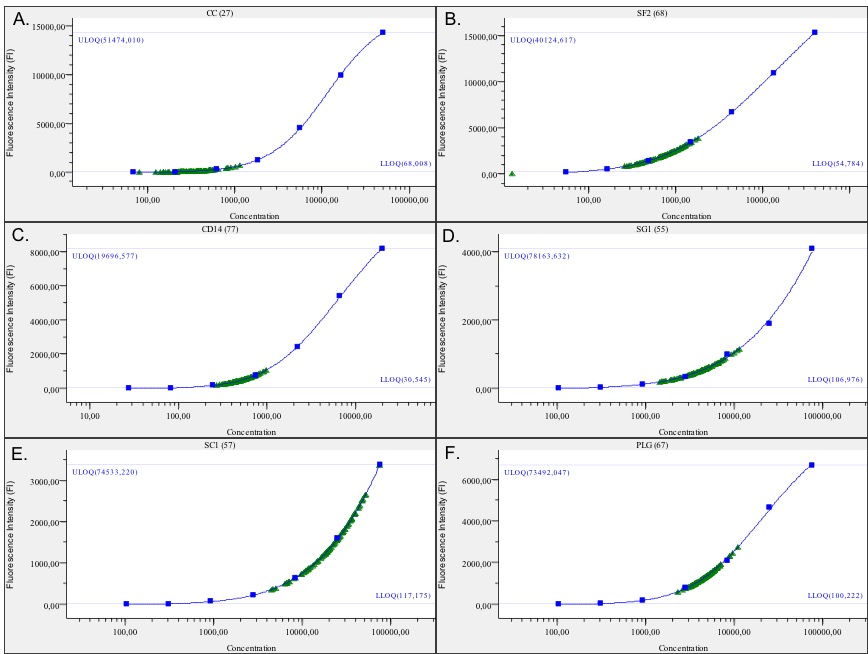
**

**Supplemental figure 3. Calibration lines bioplex 200.** Calibration lines for all six proteins; 3A. Cystatin C, 3B Serpin F2, 3C CD15, 3D Serpin G1, 3E Serpin C1 and 3F Plasminogen.


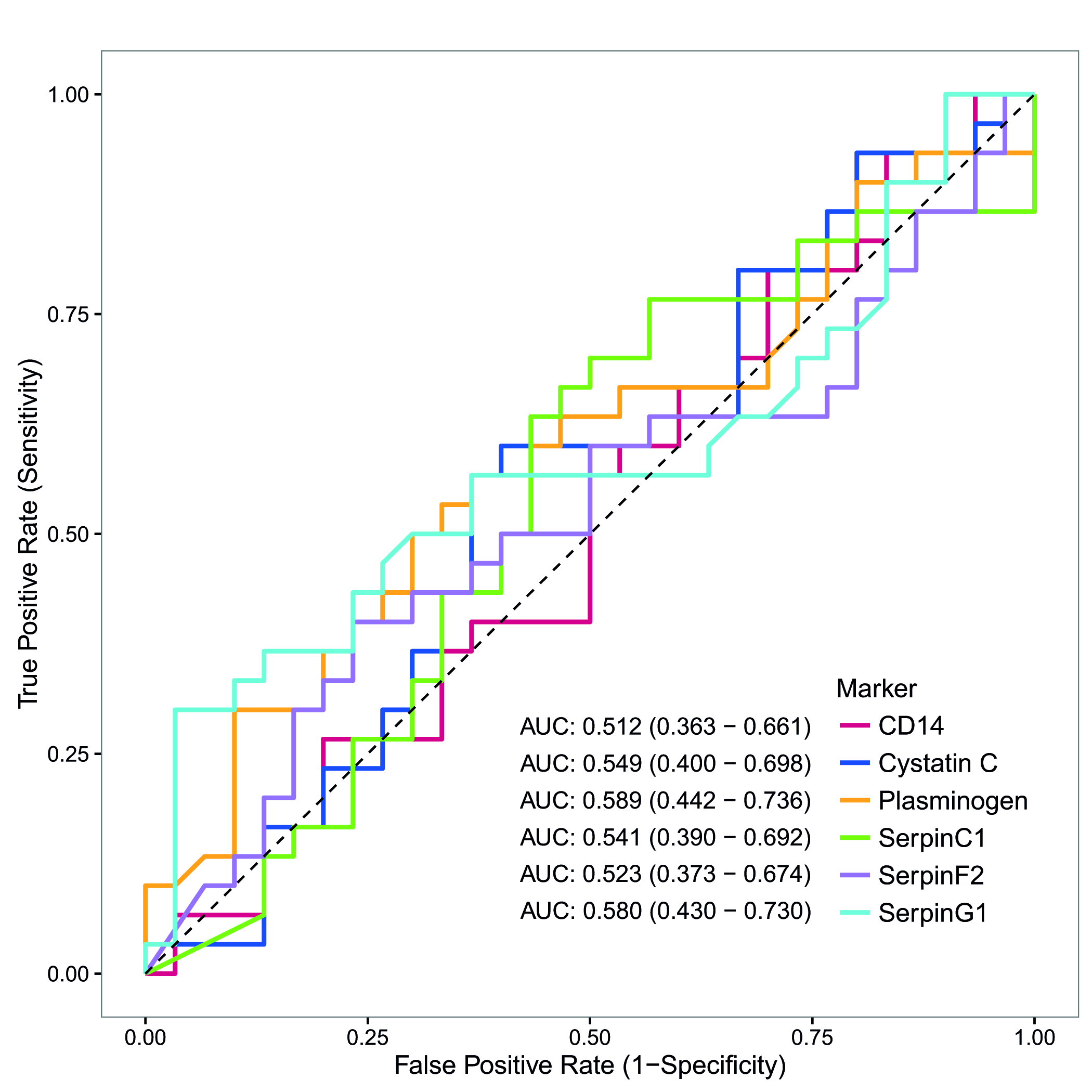


Supplemental Figure 4 ROC plasma proteins
Plasma levels of selected proteins and their diagnostic ability with AUC and 95% confidence interval

**
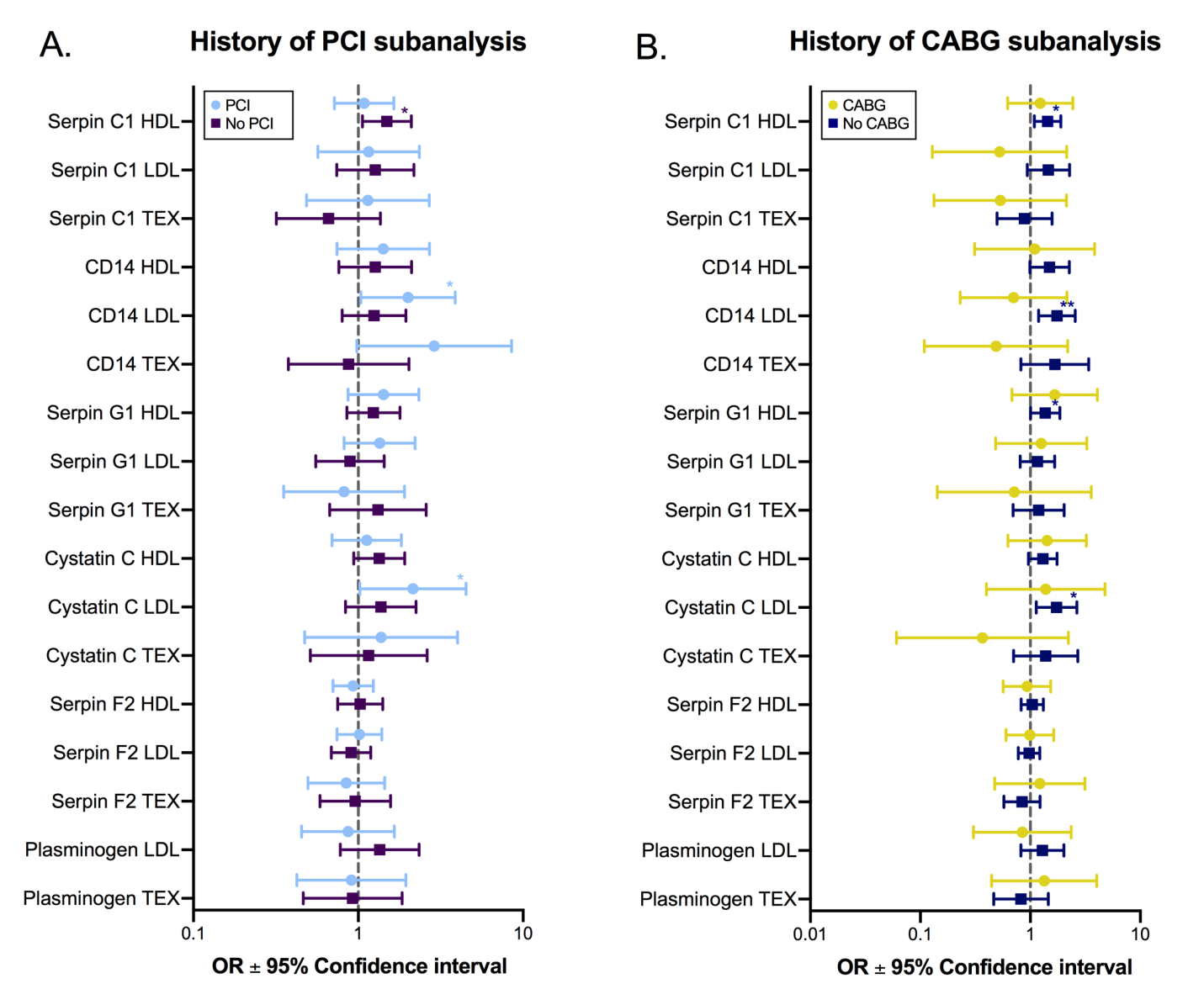
**

**Supplemental Figure 5. Forestplot of subanalysis on A. history of PCI and B. CABG**
Horizontal bars indicate adjusted* odds ratios and corresponding 95% CI on ischemia. Biomarkerlevels are logtransformed and standardized per synthetic vesicle. Original assay units are pg/ml. *Adjusted for: age, hypertension, smoking, hypercholesterolemia, diabetes mellitus and coronary artery disease. PCI = Percutaneous Coronary Intervention. CABG = Coronary Artery Bypass Graft. *Indicates P value <0.05, **P <0.01, ***P<0.001
